# Transsplenic portal vein recanalization and direct intrahepatic portosystemic shunt placement to optimize liver transplantation

**DOI:** 10.1186/s42155-019-0096-7

**Published:** 2020-01-08

**Authors:** Osman Ahmed, Abhijit L. Salaskar, Steven Zangan, Anjana Pillai, Talia Baker

**Affiliations:** 1 Section of Interventional Radiology, Department of Radiology, University of Chicago, Chicago, IL 60637 USA; 2 Department of Interventional Radiology, Amita Saint Francis Hospital, Evanston, IL 60202 USA; 3 Department of Gastroenterology, Section of Hepatology, University of Chicago, Chicago, IL 60637 USA; 4 Department of Surgery, Section of Transplant Surgery, University of Chicago, Chicago, IL 60637 USA

**Keywords:** Portal vein recanalization, PVR, Direct intrahepatic portosystemic shunt, DIPS, Portal vein occlusion, Hepatic vein occlusion, Budd Chiai syndrome

## Abstract

**Background:**

Percutaneous trans-splenic portal vein recanalization (PVR) has been reported for facilitation of transjugular intrahepatic portosystemic shunts (TIPS), however has not been applied to patients undergoing direct intrahepatic portosystemic shunt (DIPS). We report the utilization of trans-splenic-PVR with DIPS creation in a patient with chronic portal and hepatic vein occlusions undergoing liver transplantation evaluation.

**Case presentation:**

A 48-year-old male with decompensated alcoholic cirrhosis complicated by refractory ascites, hepatic encephalopathy, and variceal bleeding underwent CT that demonstrated chronic occlusion of the hepatic veins (HV), extrahepatic portal vein (PV), and superior mesenteric vein (SMV). Due to failed attempts at TIPS at outside institutions, interventional radiology was consulted for portal vein recanalization (PVR) with TIPS to treat the portal hypertension and ascites and also facilitate an end-to-end PV anastomosis at transplantation. After an initial hepatic venogram confirmed chronic HV occlusion, a DIPS with trans-splenic PVR was planned. The splenic vein was accessed under sonographic guidance using a micropuncture set and subsequently upsized to a 6 French sheath over a stiff guidewire. A splenic venogram via this access confirmed occlusion of the PV with drainage of the splenic vein (SV) through gastric varices. The thrombosed PV was then recanalized and angioplastied to restore PV flow via the transsplenic approach. A transjugular liver access kit with a modified 21-gauge needle was advanced into the IVC through the internal jugular vein (IJV) sheath and directed towards the target snare in PV. The needle was used to subsequently puncture the PV through the caudate lobe and facilitate placement of a wire into the SV. The initial portosystemic gradient (PSG) was 20 mmHg. The IJV sheath was advanced through the hepatic parenchymal tract into the main-PV and a stent-graft was placed across the main PV and into the IVC. A portal venogram demonstrated brisk blood flow through the DIPS, resolution of varices and a PSG of 8 mmHg. One month after the procedure, the patient had a significant reduction in ascites and MELD-NA score. Patient is currently listed and awaiting transplantation.

**Conclusions:**

In the setting of chronically occluded portal and hepatic veins, trans-splenic PVR DIPS may serve as an effective bridge to liver transplantation by facilitating an end to end portal vein anastomosis.

## Background

The presence of hepatic vein (HV) occlusion poses challenges for patients with decompensated liver cirrhosis being considered for transjugular intrahepatic portosystemic shunts (TIPS). Such cases can be treated by recanalization of HVs with angioplasty and/or stenting prior to performing TIPS. Alternatively, direct intrahepatic portosystemic shunt (DIPS) creation from the IVC to portal vein can also be considered. DIPS has been shown in literature to have equivalent five-year survival rates and clinical outcomes to HV recanalization with TIPS in patients with Budd-Chiari syndrome (Mukund et al. 2018). Furthermore, DIPS may be preferred in such scenarios as it mitigates the risk of HV re-occlusion and the increased technical complexity for subsequent liver transplantation.

A critical step for DIPS creation is obtaining needle access into the portal vein (PV). Prior studies have reported techniques for accessing the PV including fluoroscopic “gun sight” (Haskal et al. 1996), transabdominal ultrasound (Boyvat et al. 2006), or intracardiac echocardiogram (i.e. intravascular ultrasound) (Petersen and Clark 2008). Creation of a DIPS is however dependent on the presence of a patent PV that can be safely targeted using these described techniques. Recently, literature has cited the use of a percutaneous trans-splenic approach to perform portal vein recanalization (PVR) and facilitate TIPS placement (Thornburg et al. 2016). To the authors’ knowledge however, the feasibility of this technique has not been applied to patients requiring DIPS. We report the utilization of trans-splenic PVR with DIPS placement in a patient with both chronic portal and hepatic vein occlusion undergoing transplantation evaluation.

## Case presentation

**Fig. 1:**
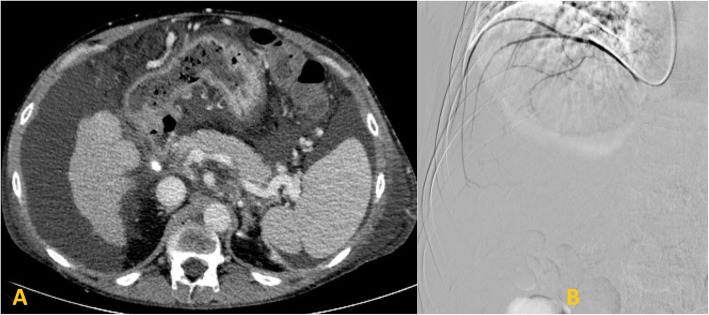
**a**: CT abdomen with contrast demonstrating occlusion of the extra-hepatic PV with cirrhotic morphology of liver, ascites and gastric varices. **b**: Venogram demonstrating non-opacification of any hepatic veins. Only a right inferior phrenic vein was selected and opacified

**Fig. 2:**
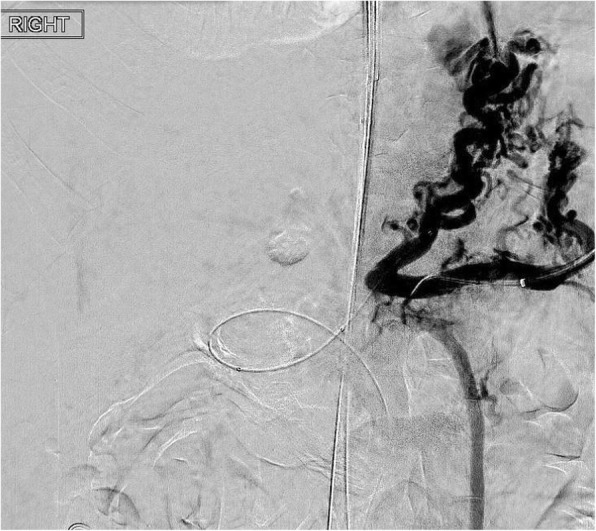
Splenic venogram demonstrating drainage of splenic vein into gastric varices and non visualization of portal vein

**Fig. 3:**
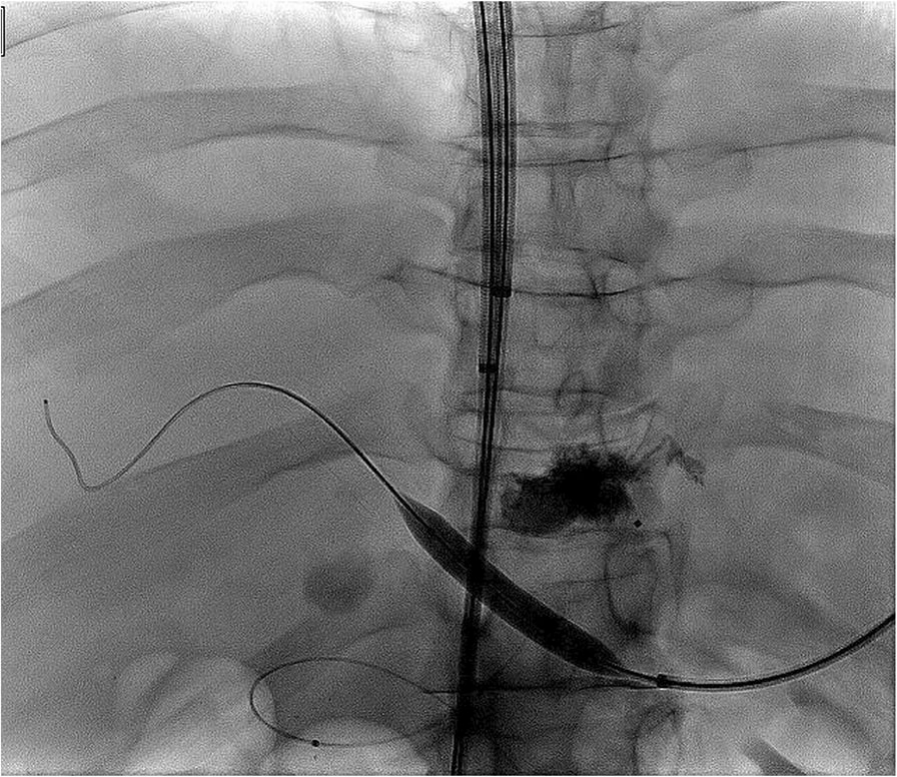
Fluoroscopic image showing balloon angioplasty of recanalized portal vein using a transsplenic approach

**Fig. 4:**
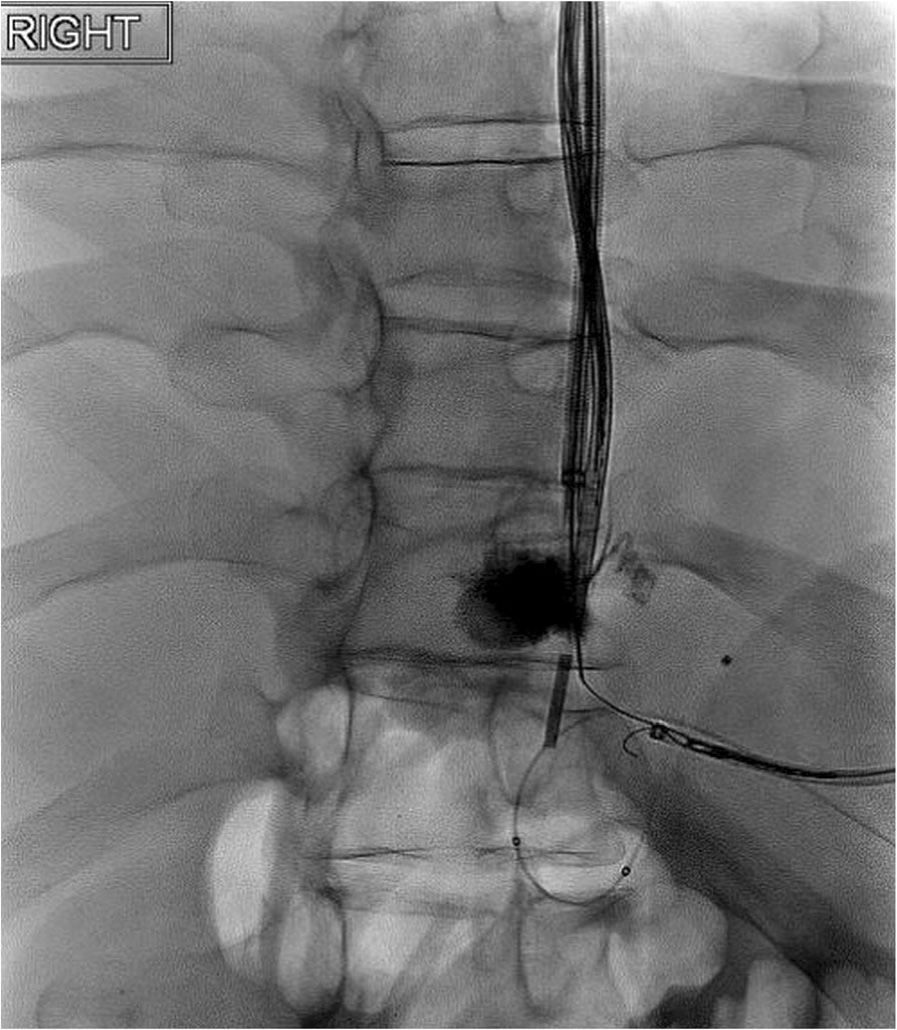
Fluoroscopic image showing a V18 wire (which was introduced through the Chiba needle) was then snared within the PV

**Fig. 5:**
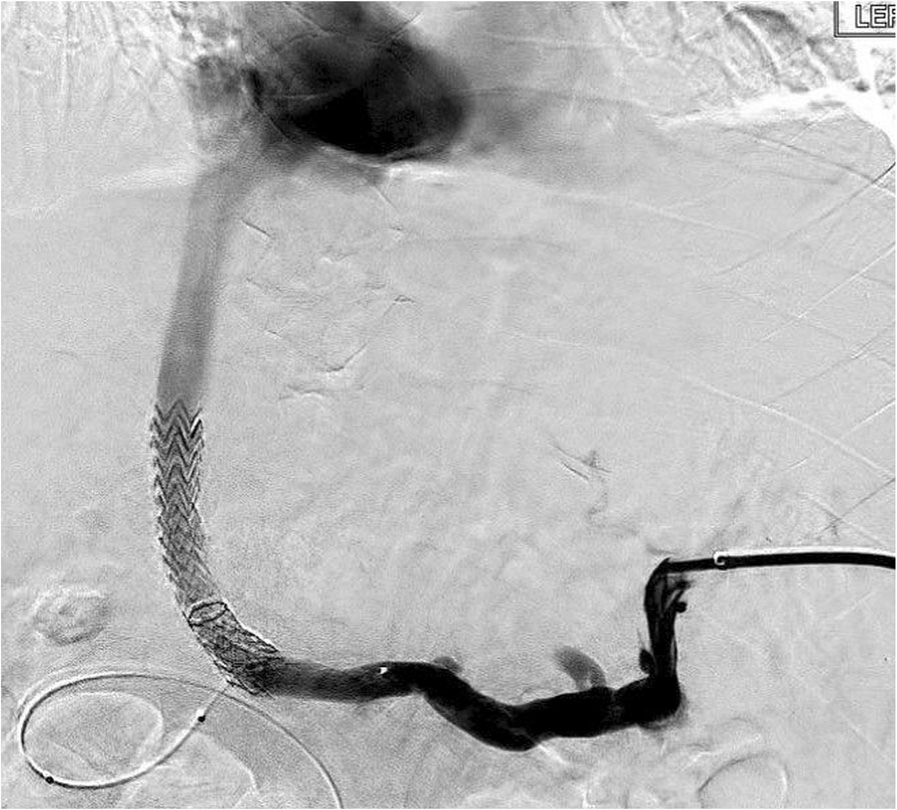
Splenic venogram showing a deployed Viatorr CX stent within PV, improved caliber of the PV and SV and resolution of varices

The patient was a 48-year-old male with decompensated alcoholic cirrhosis complicated by refractory ascites, hepatic encephalopathy, and variceal bleeding. He was admitted to our institution with worsening fluid overload and a leaking umbilical hernia. He underwent evaluation and listing for liver transplantation with a MELD-NA score of 18. Two prior attempts at TIPS had failed at outside institutions. A CT demonstrated cirrhotic morphology of the liver, stigmata of portal hypertension, as well as chronic occlusion of the HVs, extrahepatic PV (diameter < 1 mm), and distal superior mesenteric vein (SMV). **(**Figure 1**)** interventional radiology was consulted for portal vein reconstruction in combination with TIPS to alleviate portal hypertension and ascites and to facilitate transplantation allowing direct portal- portal anastomosis. After a hepatic venogram confirmed chronic hepatic vein occlusion, a multidisciplinary discussion ensued during which time an attempt at DIPS with trans-splenic PVR was decided upon. The patient was scheduled for the procedure with interventional radiology under general anesthesia. Ultrasound guidance was used to allow two 10 French sheaths to be placed into the intra-hepatic IVC via the right internal jugular vein (IJV). A percutaneous trans-splenic access into the main splenic vein was subsequently obtained by puncturing a peripheral splenic venous branch with a micropuncture set (Cook; Bloomington, IN) under sonographic guidance. The access was exchanged for a 6 Fr sheath over a stiff guidewire. A splenic venogram was performed, confirming chronic occlusion of the PV with primary drainage of the SV through gastric varices (Fig. 2). Next, the thrombosed PV was recanalized with the use of an angiographic catheter and hydrophilic wire and consequently angioplastied with an 8 mm balloon to restore its lumen and re-establish PV flow **(**Fig. 3**)**. After the PV was reconstructed, a snare was then placed into the main PV and used as a target for DIPS creation. A Roche-Uchida transjugular liver access kit with a modified 65-in. 21-gauge needle was advanced into the IVC through the sheath within the IJV and directed towards the PV. An intravascular echo probe was advanced into the IVC through the other IJV sheath and used for real time sonographic guidance of the needle puncture from the IVC to the snare in the reconstructed PV. The needle was then used to subsequently puncture the PV through the caudate lobe and facilitate placement of a wire into the SV (Fig. 4). An initial portosystemic gradient was calculated to be 20 mmHg. Following pre-dilation with an 8 mm balloon, the IJV sheath was then advanced through the hepatic parenchymal tract into the main PV. An 8–10 mm × 5 cm adjustable stent-graft was finally placed across the main PV and into the IVC to create the DIPS. The stent was dilated and angioplastied with an 8 mm balloon. A portal venogram demonstrated brisk blood flow through the DIPS and resolution of varices. A post-DIPS portosystemic gradient measured 8 mmHg. Residual narrowing and scarring within the SV was also treated with 8 mm balloon angioplasty to optimize flow through the DIPS (Fig. 5). Completion splenic venogram demonstrated brisk flow from the SV into the DIPS stent and into the IVC. Completion splenic venogram demonstrated brisk flow from the SV into the DIPS stent and into the IVC. The splenic tract was embolized with a detachable plug in addition to two 6 mm pushable coils. The patient tolerated the procedure well and remained hemodynamically stable throughout. He was initiated on anti-platelet therapy with Clopidogrel the following day for 3 months duration. At 1 month, the patient had a significant reduction in ascites with concomitant decrease in size of his ventral hernia. The MELD-NA score at this time was reduced to 12. He is currently listed but has not yet undergone transplantation.

## Conclusion

Trans-splenic PVR has been described as an effective method for restoring patency to chronically thrombosed PVs in patients with decompensated liver failure awaiting liver transplant. (Thornburg et al. 2016) As DIPS is a preferred option for patients with chronic HV occlusion, re-establishing flow in the PV and creating a target for percutaneous access remains a critical step. For this reason, in patients with both chronic PV and HV occlusion, trans-splenic PVR DIPS may represent a unique solution for creating a percutaneous portosystemic shunt. After DIPS, the restored blood flow and improved outflow can maintain PV patency and allow for residual clot resolution in the event of partial PVR. Pretransplant portal vein thrombosis is associated with increased posttransplant morbidity and mortality (Ghabril et al. 2016) Thromboendovenectomy when feasible has been advocated in these patients. However, in patients with complete portal vein thrombosis where this technique is typically not successful, a number of alternative techniques have been attempted including caval transposition, portal arterialization, and multivisceral transplantation often with discouraging results. Trans-splenic PVR TIPS has previously been shown to have very high technical success rates (Salem et al. 2015), potentiating successful end to end portal reconstruction during liver transplant with excellent post-transplant patency rates. Similarly, in more complex patients with both thrombosed PVs and HVs, trans-splenic PVR DIPS can serve as an effective bridge to transplantation and may also facilitate liver transplantation allowing for end to end portal vein anastomosis with multiple options for hepatic venous reconstruction including side-to-side cavocaostomy or complete caval replacement.

## Data Availability

Not applicable.

## Abbreviations

DIPS: Direct Intrahepatic Portosystemic Shunt
HV: Hepatic vein
IJV: Internal jugular vein
IVC: Inferior vena cava
MELD-NA: Model for End stage Liver Disease – Sodium
PV: Portal Vein
PVR: Portal vein recanalization
SMV: Superior mesenteric vein
SV: Splenic vein
TIPS: Transjugular Intrahepatic Portosystemic Shunt
